# The influence of psychosocial factors on human behaviour in emergency

**DOI:** 10.3402/ejpt.v4i0.22989

**Published:** 2013-10-30

## XIII ESTSS Conference: “Trauma and its clinical pathways: PTSD and beyond”, Bologna, June 2013 ORAL, JUNE 9 HALL LIZ

## Trauma e perdita

### L'influenza dei fattori psicosociali nel comportamento umano in emergenza   10:30–10:45

#### Elisa Saccinto^1,2^, Carles Pérez-Testor^1^ and Luca Pietrantoni^2^

^1^Blanquerna Facultat de Psicologia, Ciències de l'Educació i de l'Esport, Universitat Ramon Llull, Barcelona, Spain; ^2^Dipartimento di Psicologia, Alma Mater Studiorum Universitá di Bologna, Bologna, Italia

Lo scopo di questo studio è esplorare l'effetto di alcuni fattori psicosociali sul comportamento umano durante una situazione d'emergenza. In accordo con le osservazioni della teoria dell'Attaccamento Sociale nei disastri (Mawson 2005, 2007), le persone che si trovano in pericolo sperimentano sentimenti di attaccamento verso persone o luoghi familiari. Questi sentimenti hanno un effetto rassicurante, riducendo o ritardando i comportamenti di evacuazione. I partecipanti allo studio sono 173 superstiti di diverse situazioni d'emergenza, tra questi 42.4% sono uomini e 57.6% donne con un'età media di 32.52 anni (DS = 13.68). I risultati hanno evidenziato un'associazione negativa tra l'essere con persone familiari e il ritardo nel cominciare l'evacuazione, mentre non è emersa una relazione significativa tra la presenza di persone familiari e l'essere nella propria casa durante l'emergenza e i comportamenti di evacuazione. I risultati hanno inoltre rilevato associazioni significative tra la risposta emotiva del partecipante e il luogo: i partecipanti che si trovavano nella propria abitazione durante la situazione di pericolo hanno sperimentato più sintomi di panico, maggior disagio e più elevata percezione di pericolo nei diversi momenti dell'emergenza, rispetto a coloro che erano in luoghi pubblici. Infine, la presenza di persone familiari influenza i livelli di distress alla fine dell'emergenza. Si discutono alcune implicazioni di questi risultati per migliorare i progetti di prevenzione ed educazione sul comportamento umano in emergenza e migliorare la preparazione dei cittadini.

**Bibliografia**

Mawson, A. R. (2005). Understanding mass panic and other collective responses to threat and disaster. *Psychiatry, 68*, 95–113.

Mawson, A. R. (2007). *Mass Panic and Social Attachment. The Dynamics of Human Behaviour*. Brookfield (VT): Ashgate Publishers (Eds).

### The influence of psychosocial factors on human behaviour in emergency

The aim of this study is to explore the effect of some psychosocial factors on human behaviour during an emergency situation. In agreement with the observations of the theory of social attachment in disasters (Mawson 2005, 2007), people in danger experience feelings of attachment for familiar persons and places. These feelings have a reassuring effect, reducing or delaying the behaviour of evacuation. Participants were 173 survivors of several emergency situations: 42.4% were men and 57.6% were women with mean age 32.52 years (SD = 13.68). Results evidenced a negative association between being with familiar people and delay in starting the evacuation, whereas the presence of familiar people or being at home during the danger situation were not significantly associated with evacuation. Findings also highlighted significant associations between participants’ emotional response and place: participants who were at home during the danger situation experienced more panic symptoms, distress, and perception of threat in the different moments of the evacuation in comparison with those who were in public buildings. Finally, it was concluded that the presence of familiar people had an influence on the level of distress during emergency situation. Here, we discuss some implications of these results for prevention and education programs on human behaviour during emergencies and for improving citizens’ preparedness.

